# FSP1 and histone deacetylases suppress cancer persister cell ferroptosis

**DOI:** 10.1126/sciadv.aea8771

**Published:** 2026-01-02

**Authors:** Masayoshi Higuchi, August F. Williams, Anna E. Stuhlfire, Ariel H. Nguyen, David A. G. Gervasio, Claire E. Turkal, Suejean Chon, Matthew J. Hangauer

**Affiliations:** ^1^ Department of Dermatology, School of Medicine, University of California San Diego, San Diego, CA, USA.; ^2^ Moores Cancer Center, University of California San Diego, San Diego, CA, USA.; ^3^ Altman Clinical and Translational Research Institute, University of California San Diego, San Diego, CA, USA.

## Abstract

Cancer persister cells which survive oncogene targeted therapies are sensitized to ferroptosis, but mechanistic understanding of this vulnerability remains limited. Here, we found that while levels of iron, glutathione, and various ferroptosis-suppressing enzymes vary among persister cell types, ferroptosis suppressor protein 1 (FSP1) is down-regulated in multiple persister cell types, and persister cells which survive glutathione peroxidase 4 (GPX4) inhibition rely on residual FSP1 to survive. Furthermore, persister cells which survive GPX4 inhibition down-regulate oxidative phosphorylation, a key source of mitochondrial reactive oxygen species which are required for persister cell ferroptosis. We also found that persister cell treatment with histone deacetylase inhibitors induces reactive oxygen species and sensitizes multiple persister cell types to GPX4 inhibition. Together, these findings reveal that FSP1 and histone deacetylases suppress persister cell ferroptosis.

## Teaser

Drug-tolerant cancer persister cells can be sensitized to ferroptotic death by FSP1 or histone deacetylase inhibitors.

## INTRODUCTION

Drug-tolerant cancer persister cells survive cytotoxic drug treatments, populate minimal residual disease, undergo mutagenesis, and may contribute to acquired resistance and tumor recurrence (*1*). Therapeutic eradication of persister cells may increase the durability of responses to existing cancer treatments. Unfortunately, there are no clinically approved therapies which are intended to target persister cells. Identification of a robust persister cell drug target has been challenging because most of the known persister cell vulnerabilities are restricted to specific cell types and drug treatments (*2*–*11*). One notable exception is ferroptosis. We previously found that persister cells from multiple tumor types and treatments are vulnerable to ferroptosis which can be induced with inhibitors of the lipid hydroperoxidase glutathione peroxidase 4 (GPX4) (*3*). There are multiple drug development efforts underway targeting GPX4, but GPX4 is an essential enzyme raising toxicity concerns and thus far there have been no reported GPX4 inhibitors with potent in vivo efficacy. Also, a GPX4 inhibitor must not only achieve bioavailability, it may also need enhanced potency because ferroptosis sensitivity in vivo is diminished compared to cell culture (*12*–*14*).

Alternative approaches to inducing ferroptosis in persister cells may overcome these challenges. Unfortunately, it remains poorly understood why persister cells are selectively sensitized to ferroptosis despite a variety of factors which promote ferroptosis sensitivity being known in other contexts (*15*). It was recently postulated that persister cells use iron to survive, similar to cancer stem cells and cells undergoing epithelial to mesenchymal transition (*4*, *16*–*18*), and that this results in enhanced sensitivity to ferroptosis (*19*). However, although iron chelation protects persister cells from ferroptosis demonstrating iron is required (*3*), iron levels vary as decreased or increased iron levels have been previously measured in breast (*3*) and lung cancer persister cell models (*20*), respectively. Subcellular labile iron pools, such as within the lysosome (*21*–*23*), may also differ in persister cells and contribute to ferroptosis. Another possibility is that persister cells are deficient in antioxidant defenses. We previously found that persister cells have lower basal glutathione (GSH) levels, which was also observed in another persister cell model (*20*), and that GSH is rapidly further depleted, and reactive oxygen species (ROS) markedly increases upon GPX4 inhibition in persister but not drug naïve parental cells (*3*). However, there is relatively modest rescue from persister cell ferroptosis by GSH replenishment, indicating that low GSH may not be necessary for ferroptosis sensitivity (*3*). Together, these observations show that while cellular iron and GSH levels influence ferroptosis, neither fully explain why persister cells are sensitized to ferroptosis.

In this study, we sought to explore alternative explanations for persister cell ferroptosis sensitivity. Persister cells have been previously reported to preferentially depend on oxidative phosphorylation (OXPHOS), a major source of mitochondrial ROS, instead of glycolysis metabolism (*24*–*26*). Here, we found that persister cell mitochondrial ROS is essential for ferroptosis susceptibility and that persister cells which survive ferroptosis have decreased OXPHOS. We also found that treatment of persister cells with otherwise nontoxic concentrations of clinically available histone deacetylase (HDAC) inhibitors which increase ROS further sensitizes persister cells, but not parental cells, to ferroptosis. Also, persister cells depend on ferroptosis suppressor protein 1 (FSP1), which is down-regulated in persister cells, to survive GPX4 inhibition. Therefore, HDAC inhibitors or FSP1 inhibitors may be combined with GPX4 inhibitors to selectively enhance persister cell ferroptosis. These findings provide insight into why cancer persister cells are sensitized to ferroptosis and reveal potential combinatorial treatment strategies to enhance persister cell elimination.

## RESULTS

### Mitochondrial ROS contributes to persister cell sensitivity to ferroptosis

**Fig. 1.**
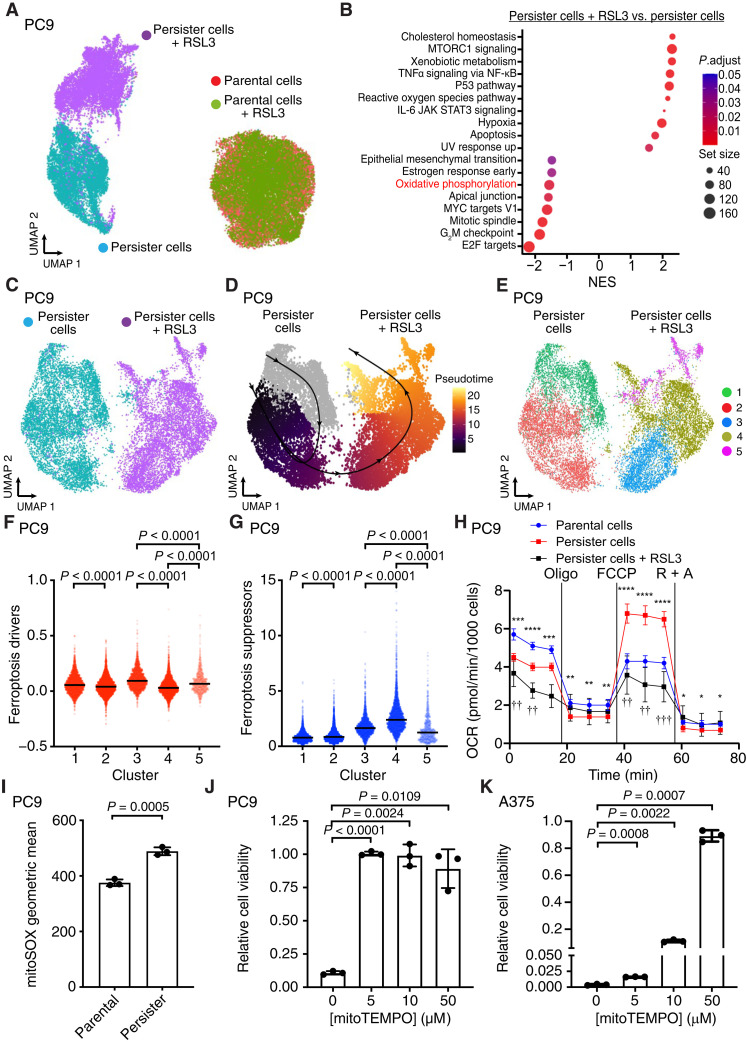
OXPHOS contributes to ferroptosis sensitization in cancer persister cells. (**A**) UMAP of PC9 parental and persister cells treated with or without 1 μM RSL3 for 24 hours. (**B**) Enriched Hallmarks gene sets in persister cells treated with or without RSL3. Positive normalized enrichment score (NES) values indicate enrichment in RSL3-treated persister cells. TNFα, tumor necrosis factor–α; NF-κB, nuclear factor κB; IL-6, interleukin-6; JAK, Janus kinase; STAT3, signal transducer and activator of transcription 3; UV, ultraviolet. (**C**) UMAP of PC9 persister cells treated with and without RSL3. (**D**) Pseudotime analysis of PC9 persister cells treated with and without RSL3. Solid black line indicates the estimated trajectory across cell states. (**E**) UMAP of PC9 persister cells treated with and without RSL3 colored by cluster. (**F**) Ferroptosis driver gene set signature score across clusters in (E). (**G**) Ferroptosis suppressor gene set signature score across clusters in (E). [(F) and (G)] *P* values calculated with Mann-Whitney test. (**H**) PC9 parental, persister, and persister cells treated with 500 nM RSL3 for 24 hours were analyzed for oxygen consumption rate (OCR). A total of 1.25 μM oligomycin (Oligo), 1 μM carbonyl cyanide *p*-trifluoromethoxyphenylhydrazone (FCCP), and 1 μM rotenone plus 1 μM antimycin A (R + A). *n* = 3 biological replicates; mean ± SEM is shown; *P* values calculated between parental and persister cell conditions (stars) or between persister cells and RSL3-treated persister cells (crosses) using two-tailed Student’s *t* test. *P < 0.05, **P < 0.01, ***P < 0.001, ****P < 0.0001, ††*P* < 0.01, and †††*P* < 0.001. (**I**) PC9 parental and persister cells derived from 2.5 μM erlotinib analyzed for mitochondrial ROS. Par, parental; pers, persister. (**J** and **K**) PC9 persister cells derived from 2.5 μM erlotinib (J) and A375 persister cells derived from 250 nM dabrafenib and 25 nM trametinib (K) in combination with mitoTEMPO were treated with 500 nM RSL3 for 24 hours. Viability was normalized to the respective mitoTEMPO-treated persister cells without RSL3 treatment. [(I) to (K)] *n* = 3 biological replicates; mean ± SD is shown; *P* values calculated with two-tailed Student’s *t* test.

We reasoned that analysis of the subpopulation of persister cells which survive partially lethal GPX4 inhibition may reveal features which functionally govern their sensitivity to ferroptosis. We performed single-cell RNA sequencing (scRNA-seq) on epidermal growth factor receptor (EGFR) mutant PC9 non–small cell lung cancer persister cells, derived from 10 days of treatment with EGFR inhibitor erlotinib, which were subsequently treated with GPX4 inhibitor RAS-selective lethal 3 (RSL3) for 24 hours resulting in partial death and effectively purifying surviving persister cells. Persister cells which survived RSL3 treatment were nearly completely separated from persister cells which were not treated with RSL3 on a Uniform Manifold Approximation and Projection (UMAP) plot, indicating that surviving cells were altered by RSL3 exposure (Fig. 1A). In contrast, drug naïve PC9 parental cells treated with the same concentration and duration of RSL3, which is nontoxic to parental cells, entirely overlapped untreated parental cells, indicating that GPX4 inhibition has a minimal effect on parental cells (Fig. 1A). These observations are consistent with our prior findings that persister cells are sensitized to ferroptosis relative to parental cells (*3*).

Gene set enrichment analysis of persister cells revealed that pathways known to protect from ferroptosis were enriched among persister cells, which survived RSL3 treatment including cholesterol homeostasis (*27*), mechanistic target of rapamycin (mTOR) signaling (*28*), xenobiotic (GSH) metabolism (*29*), tumor necrosis factor–α signaling via nuclear factor κB (*30*), and p53 signaling (Fig. 1B and tables S1 and S2) (*31*). The epithelial-to-mesenchymal gene set was also down-regulated in cells surviving RSL3 treatment, consistent with prior reports that mesenchymal cancer cells are sensitized to ferroptosis (Fig. 1B) (*4*). Furthermore, a YAP gene set signature was depleted among persister cells surviving RSL3 treatment (fig. S1 and table S2). It was previously reported that loss of YAP signaling upon cell contact–induced activation of the Hippo pathway protects cells from ferroptosis (*13*). This raised the possibility that low cell density may explain persister cell sensitivity to ferroptosis. To test this, we generated “high-density” PC9 persister cells and BRAF and MAPK kinase inhibitor–derived BRAF V600E A375 melanoma persister cells by treating with lower drug concentrations for 10 days, resulting in fully confluent persister cells, and found that high-density persister cells remain selectively sensitized to ferroptosis versus density-matched parental cells of equal or lower confluency (fig. S2). Therefore, persister cell sensitization to ferroptosis is not merely due to low cell density.

Pseudotime analysis of the scRNA-seq data revealed a trajectory from persister cells, which were not treated with RSL3 toward cells that survived RSL3 treatment (Fig. 1, C to E). Persister cells which survived RSL3 treatment were separated into three clusters. Along the pseudotime trajectory, the first RSL3-treated cluster (cluster 3) is characterized by relatively high expression of a ferroptosis driver gene set signature and lower ferroptosis suppressor signature compared to the other RSL3-treated clusters (*32*), suggesting that this cluster contains relatively ferroptosis-sensitive cells (Fig. 1, D to G). In contrast, the next RSL3-treated persister cell cluster along the trajectory (cluster 4) has lower expression of the ferroptosis driver gene set and higher expression of the ferroptosis suppressor gene set, suggesting that this population is more protected from ferroptosis (Fig. 1, D to G). The third RSL3-treated cluster (cluster 5) has relatively few cells and exhibited a high ferroptosis driver signature and low ferroptosis suppressor signature, suggesting this is also a ferroptosis-sensitive population (Fig. 1, E to G, and tables S3 and S4).

Among the gene sets differentially expressed in ferroptosis-resistant cluster 4 versus the other two RSL3-treated clusters, we observed decreased expression of the Hallmarks OXPHOS gene set (tables S3 and S4). Given that the bulk population of RSL3-treated persister cells also displayed decreased expression of the OXPHOS gene set (Fig. 1B), we hypothesized that OXPHOS, a persister cell dependency and ROS source (*24*, *25*), contributes to ferroptosis sensitization of persister cells. We first investigated the levels of OXPHOS across parental, persister, and persister cells treated with RSL3. Previous studies reported increased OXPHOS in persister cells, and consistent with this, we found that persister cells have an increased maximal respiration capacity compared to parental cells (Fig. 1H) (*24*–*26*). Furthermore, we found that electron transport chain inhibitor treatment, which inhibits persister cell OXPHOS, was highly toxic to persister cells (fig. S3, A to F). Also, consistent with the decreased OXPHOS gene set expression in persister cells treated with RSL3 (Fig. 1B), we observed lower OXPHOS in this population compared to non–RSL3-treated persister cells, suggesting that higher OXPHOS promotes ferroptosis sensitivity (Fig. 1H).

OXPHOS could sensitize persister cells to ferroptosis through several mechanisms including adenosine triphosphate (ATP) production (*33*, *34*), ubiquinol replenishment (*35*), or mitochondrial ROS generation (*36*). Increased ATP levels can block ferroptosis-protecting AMP-activated protein kinase (AMPK) activity (*37*). However, we did not observe significantly increased ATP in persister cells (fig. S4A), and neither parental nor persister cells display phosphorylated AMPK, indicating a lack of AMPK activation (fig. S4B). In addition, treatment with the mitochondrial uncoupler carbonyl cyanide *p*-trifluoromethoxyphenylhydrazone (FCCP) to block ATP production, which selectively kills persister cells consistent with persister cell OXPHOS dependency (fig. S4, C and D), did not protect persister cells from GPX4 inhibition (fig. S4E). We also found that cotreatment with the dihydroorotate dehydrogenase (DHODH) inhibitor BAY2402234, which blocks ubiquinone reduction to ubiquinol in mitochondria, did not further sensitize persister cells (fig. S4, F and G). We next tested whether OXPHOS sensitizes persister cells to ferroptosis through the generation of mitochondrial ROS. Consistent with this possibility, we observed an increase in mitochondrial ROS in persister cells (Fig. 1I and fig. S4H). Furthermore, treatment with the mitochondrial-targeted antioxidant mitoTEMPO protected persister cells from GPX4 inhibition, demonstrating that mitochondrial ROS contributes to persister cell ferroptosis sensitivity (Fig. 1, J and K, and fig. S4I). Together, these data are consistent with OXPHOS-promoting persister cell ferroptosis sensitization through the production of mitochondrial ROS.

### FSP1 protects persister cells from GPX4 inhibition

**Fig. 2.**
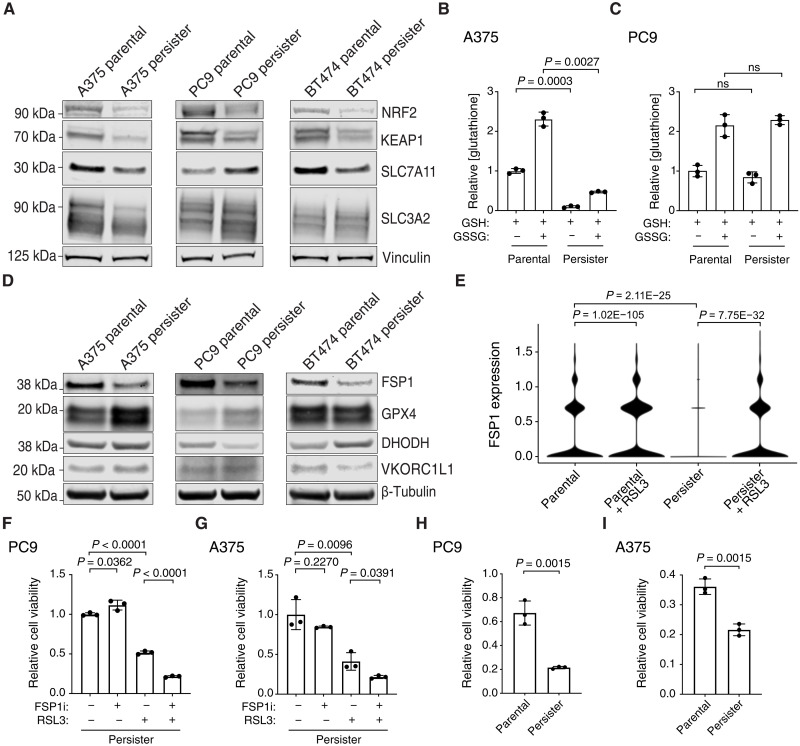
Persister cells have variable antioxidant deficiencies and depend on FSP1 to survive GPX4 inhibition. (**A**) Parental and persister cells analyzed for NRF2, KEAP1, and system x_c_^−^ components SLC7A11 and SLC3A2 expression. (**B** and **C**) A375 and PC9 parental and persister cells analyzed for reduced GSH or total GSH (GSSG) levels. (**D**) Protein expression of ferroptosis suppressor genes in parental and persister cells. (**E**) FSP1 mRNA expression in PC9 parental and persister cells treated with and without RSL3. *P* values calculated with the Wilcoxon rank sum test with Bonferroni correction. (**F** and **G**) PC9 (F) and A375 (G) persister cells cotreated with 1 μM FSP1 inhibitor, iFSP1, 50 nM RSL3, or both for 24 hours. (**H** and **I**) PC9 (H) and A375 (I) viability of parental cells and persister cells after treatment with the combination of 1 μM iFSP1 with 50 nM RSL3 for 24 hours. [(B), (C), and (F) to (I)] *n* = 3 biological replicates; mean ± SD is shown; *P* values calculated with two-tailed Student’s *t* test. ns, not significant.

We previously found that human epidermal growth factor receptor 2 (HER2)-amplified BT474 breast cancer persister cells which are sensitized to ferroptosis have decreased expression of nuclear factor E2-related factor 2 (NRF2) target genes, reflecting a disabled antioxidant state (*3*). Here, we found that NRF2 protein levels are decreased in all tested persister cell types relative to parental cells (Fig. 2A). We also found that PC9 persister cells which survive RSL3 are enriched for the NRF2 oncogenic signature gene set (fig. S1 and table S2). However, the role NRF2 plays in ferroptosis protection is controversial (*15*), and unlike BT474 persister cells, we found that despite lower NRF2 protein levels (Fig. 2A), PC9 persister cells show enrichment rather than depletion of the NRF2 gene set compared to parental cells (fig. S5 and tables S5 and S6). We also observed that NRF2-negative regulator kelch-like ECH-associated protein 1 (KEAP1) is consistently decreased rather than increased across persister cell types, reflecting potentially complex regulation (Fig. 2A). Therefore, while NRF2 activity may protect from ferroptosis, loss of NRF2 activity does not appear to universally contribute to persister cell ferroptosis sensitivity.

We previously found that BT474 persister cells exhibit decreased expression of solute carrier family 7 member 11 (SLC7A11), a component of system x_C_^−^ which is responsible for the transport of the GSH precursor cystine into the cell (*15*), and have decreased GSH levels (*3*). Here, we found that PC9 persister cells with increased xenobiotic metabolism, which is related to GSH function, were selected for among persister cells which survived RSL3 treatment (Fig. 1B). Together with our prior observation that GSH is rapidly depleted in BT474 persister but not parental cells upon GPX4 inhibition and that *N*-acetyl cysteine treatment partially rescues from ferroptosis (*3*), these data support that GSH counteracts ferroptosis in persister cells. However, relative levels of system x_C_^−^ components SLC7A11 and solute carrier family 3 member 2 (SLC3A2) proteins are variable across persister cell types, and while A375 persister cells have reduced GSH levels compared to parental cells, similar to BT474 persister cells, PC9 persister cells do not (Fig. 2, A to C). Therefore, while GSH protects from ferroptosis, decreased persister cell GSH is not a general cause of cancer persister cell ferroptosis sensitivity.

We next investigated the protein levels of select ferroptosis-suppressing enzymes including GPX4 (*38*), FSP1 (*39*, *40*), DHODH (*35*), and vitamin K epoxide reductase complex subunit 1-like 1 (VKORC1L1) (*41*) to determine whether loss of any of these factors could explain the persister cell dependence on GPX4. FSP1 protein expression was decreased across all persister cell models, while GPX4, DHODH, and VKORC1L1 levels were variable (Fig. 2D). Furthermore, while FSP1 mRNA expression was decreased in PC9 persister cells compared to parental cells, it increased in persister cells which survive GPX4 inhibition (Fig. 2E). On the basis of this, we hypothesized that persister cells which survive GPX4 inhibition may become reliant on FSP1 to survive. We therefore tested whether GPX4 inhibitor–treated persister cells are sensitized to FSP1 inhibition. While FSP1 inhibition was previously reported to enhance GPX4 inhibitor–induced ferroptosis in parental cancer cells (*39*, *42*, *43*), we found that nontoxic concentrations of the FSP1 inhibitor iFSP1 sensitized persister cells to GPX4 inhibitor more than parental cells (Fig. 2, F to I, and fig. S6). Therefore, FSP1 protects persister cells from GPX4 inhibition, and combining inhibition of FSP1 and GPX4 selectively enhances persister cell ferroptosis.

### HDAC inhibitors panobinostat and vorinostat synergize with GPX4 inhibition to induce persister cell death

**Fig. 3.**
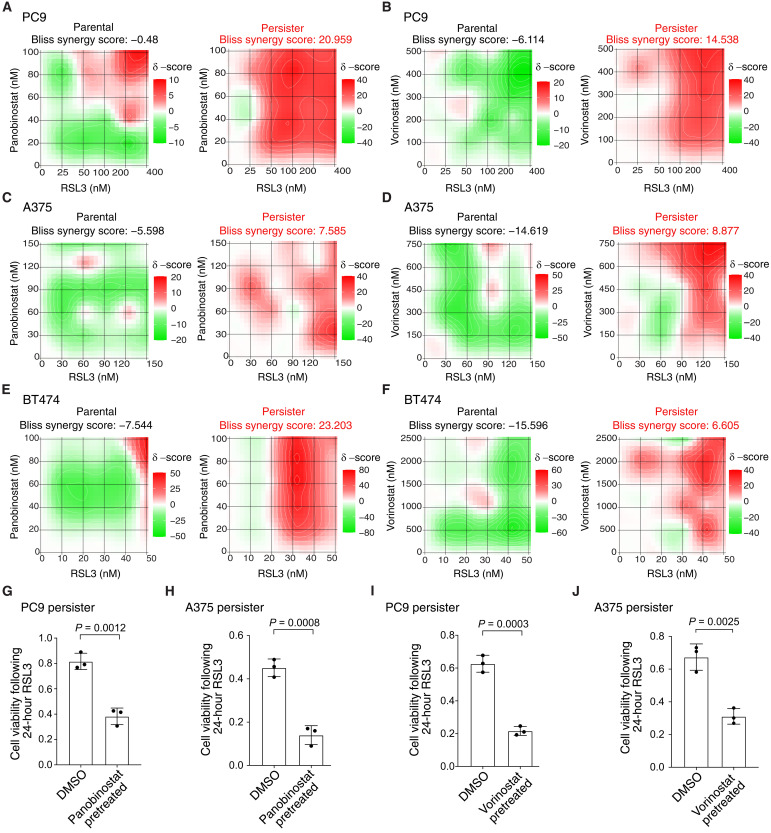
HDAC inhibition synergizes with GPX4 inhibition to selectively kill persister cells. (**A** to **F**) Synergy heatmaps between GPX4 inhibitor RSL3 and HDAC inhibitors panobinostat and vorinostat following 24-hour cotreatment. Bliss synergy score calculated with SynergyFinder 3.0. Red color and positive scores indicate synergy, and green color and negative scores indicate buffering. [(A) and (B)] PC9 parental cells and persister cells derived from 50 nM erlotinib. [(C) and (D)] A375 parental cells and persister cells derived from 10 nM dabrafenib with 1 nM trametinib. [(E) and (F)] BT474 parental cells and persister cells derived from 2 μM lapatinib. (**G** to **J**) Prederived PC9 or A375 persister cells were treated for 48 hours with a nontoxic concentration of HDAC inhibitor (see fig. S7), rinsed, and then treated with RSL3 for 24 hours while maintained under targeted therapy treatment. Data normalized to untreated persister cells. Concentration and HDAC inhibitor used were as follows: (G) 7.5 nM panobinostat, (H) 5 nM panobinostat, (I) 100 nM vorinostat, and (J) 1 μM vorinostat. RSL3 concentrations used were as follows: (G) 150 nM, (H) 100 nM, (I) 150 nM, and (J) 80 nM. *n* = 3 biological replicates; mean ± SD is shown; *P* values calculated with two-tailed Student’s *t* test.

HDAC inhibitors were recently shown to enhance ferroptosis in combination with inhibitors of system x_C_^−^ or GPX4 in other contexts (*44*–*51*). We therefore explored whether HDAC inhibition also promotes persister cell ferroptosis. We found that clinically used pan-HDAC inhibitors panobinostat and vorinostat synergize with GPX4 inhibitors to induce cell death in lung, melanoma, and breast cancer persister cell models but not in parental cells (Fig. 3, A to F, and fig. S7A). We also found synergy between RSL3 and another epigenetic modifier, bromodomain-containing protein 4 inhibitor JQ1 (fig. S7B). Furthermore, pretreatment with a nontoxic concentration of either HDAC inhibitor sensitized persister cells, but not parental cells, to subsequent GPX4 inhibitor treatment (Fig. 3, G to J, and fig. S7, C to H). Therefore, modulation of persister cell epigenetic states can sensitize to ferroptosis.

**Fig. 4.**
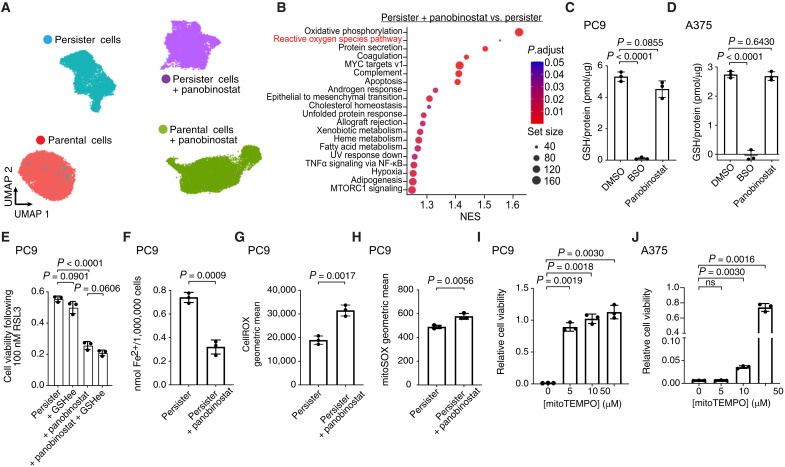
HDAC inhibitors induce persister cell oxidative stress to sensitize persister cells to ferroptosis. (**A**) UMAP of PC9 parental and persister cells treated with or without HDAC inhibitor panobinostat for 48 hours. (**B**) Enriched Hallmarks gene sets between persister cells treated with and without panobinostat. Positive NES values indicate gene sets enriched in persister cells treated with panobinostat. (**C** and **D**) Treatment with panobinostat does not decrease GSH levels in PC9 (7.5 nM panobinostat) or A375 (5 nM panobinostat) persister cells. Buthionine sulfoximine (BSO; 1 mM) was used as a positive control for GSH depletion. (**E**) Ferroptosis sensitization of PC9 persister cells from treatment with panobinostat is not inhibited by GSH ethyl ester (GSHee, 1 mM). (**F**) PC9 persister cell treatment with panobinostat decreases rather than increases intracellular iron. (**G** and **H**) Panobinostat treatment of PC9 persister cells increases total cellular ROS (G) and mitochondrial ROS (H). (**I** and **J**) PC9 persister cells derived from 2.5 μM erlotinib (I) and A375 persister cells derived from 250 nM dabrafenib and 25 nM trametinib (J) were cotreated with mitoTEMPO and were treated with 7.5 and 5 nM panobinostat, respectively, for 48 hours with and without 500 nM RSL3 for 24 hours. Viability was normalized to the viability of targeted therapy with mitoTEMPO and panobinostat without RSL3. [(C) to (J)] *n* = 3 biological replicates; mean ± SD is shown; *P* values calculated with two-tailed Student’s *t* test.

We performed scRNA-seq on panobinostat-treated PC9 persister cells and found that, unlike RSL3 treatment which solely affects persister cell transcriptomes (Fig. 1A), panobinostat causes broad transcriptional changes to both parental and persister cells (Fig. 4A). The GSH-related gene set xenobiotic metabolism was enriched in panobinostat-treated persister cells (Fig. 4B). However, panobinostat did not affect persister cell GSH levels (Fig. 4, C and D), and addition of excess GSH did not rescue persister cells from panobinostat-induced ferroptosis sensitization (Fig. 4E). Furthermore, while the heme metabolism gene set was enriched in panobinostat-treated persister cells indicating a potential role for iron (Fig. 4B), we found that panobinostat lowered rather than increased the labile iron content in persister cells (Fig. 4F). Therefore, panobinostat treatment enhances persister cell sensitization to ferroptosis independent of GSH or iron levels.

We next explored whether panobinostat treatment induces ROS in persister cells because the ROS pathway was among the top Hallmarks gene sets enriched upon panobinostat treatment in persister cells (Fig. 4B and tables S7 and S8). Panobinostat modestly decreased persister cell OXPHOS yet increased expression of the OXPHOS Hallmarks gene set (Fig. 4B and fig. S8A) with the net effect of, consistent with prior reports of HDAC inhibition–induced ROS in cancer cells (*52*–*56*), increasing total cellular ROS and mitochondrial ROS in PC9 persister cells (Fig. 4, G and H, and fig. S8B). Furthermore, antioxidants EUK-134, nordihydroguaiaretic acid, and mitoTEMPO protected both PC9 and A375 persister cells from panobinostat-induced ferroptosis sensitization (Fig. 4, I and J, and fig. S8, C and D). Together, these data show that HDAC inhibitor–induced oxidative stress sensitizes persister cells to ferroptosis.

## DISCUSSION

We previously reported that cancer persister cells are sensitized to ferroptosis (*3*). Since that time, accumulating data have reinforced this finding (*1*, *20*, *22*, *57*–*61*), yet there has been minimal progress in understanding why ferroptosis is an emergent persister cell vulnerability. Furthermore, a potent chemical inducer of ferroptosis with strong efficacy and minimal toxicity in vivo has yet to be developed. An improved mechanistic understanding of persister cell ferroptosis susceptibility may help identify therapeutic approaches which overcome current hurdles.

We reasoned that transcriptomic changes within persister cells which survive brief ferroptotic stress may implicate pathways which govern persister cell ferroptosis sensitivity. Consistent with ferroptosis as a persister cell–selective vulnerability, we found that while persister cells which survive GPX4 inhibitor exposure are transcriptionally distinct from other persister cells, drug naïve parental cells are transcriptionally unaffected by GPX4 inhibition. Furthermore, we found that OXPHOS is decreased among persister cells which survive RSL3 treatment, indicating that mitochondrial metabolism may contribute to persister cell ferroptotic death. While we found that mitochondrial ATP production and ubiquinol replenishment did not affect persister cell ferroptosis sensitivity, persister cells exhibit elevated total cellular and mitochondrial ROS, and treatment with mitochondrial-targeted antioxidant mitoTEMPO strongly protects persister cells from ferroptosis. Given that persister cells depend on OXPHOS for survival and OXPHOS is a primary source of mitochondrial ROS (*1*, *24*, *25*, *62*), these findings suggest that persister cell ferroptosis sensitivity results at least in part from persister cell OXPHOS dependence.

In addition to elevated ROS, a disabled antioxidant program may also sensitize persister cells to ferroptosis. We previously found that HER2-amplified BT474 breast cancer persister cells have a broadly disabled antioxidant program with diminished expression of NRF2 target genes including system x_C_^−^ components and decreased levels of GSH and nicotinamide adenine dinucleotide phosphate (NADPH) (reduced form of NADP^+^) (*3*). However, upon surveying additional persister cell types, we found variable levels of antiferroptotic factors with the exception of FSP1 which was depleted in each. We also found that persister cells depend on residual FSP1 to survive GPX4 inhibitor treatment, because FSP1 inhibition sensitized persister cells to GPX4 inhibition. However, parental cells are also killed by combining FSP1 and GPX4 inhibition, although to a lesser degree than persister cells, suggesting that this combination treatment may be useful to simultaneously induce ferroptosis in both tumor cell populations.

We also found that the clinically approved pan-HDAC inhibitors panobinostat and vorinostat synergize with GPX4 inhibitors to selectively enhance persister cell ferroptosis. Also, brief nontoxic panobinostat or vorinostat pretreatment sensitizes persister but not parental cells to subsequent GPX4 inhibition, pointing toward combinatorial HDAC inhibition with GPX4 inhibition as a potential strategy to enhance persister cell ferroptosis. Although HDAC inhibition yields mixed results in solid tumors (*63*), our data suggest that if applied to minimal residual disease, HDAC inhibitors may prime persister cells for ferroptosis. HDAC inhibitor treatment has previously been shown to promote ferroptosis sensitivity in other contexts in which increased iron or GSH depletion due to SLC7A11 down-regulation was identified as a mechanism (*44*–*52*, *64*). However, we found that HDAC inhibitor decreased labile iron and did not affect GSH levels in PC9 lung cancer persister cells. Instead, HDAC inhibitors increased persister cell ROS levels, as has also been reported in other contexts (*52*–*56*), and HDAC inhibitor sensitization of persister cells to GPX4 inhibition was blocked by mitoTEMPO, indicating that ROS is required for HDAC inhibitor–promoted ferroptosis. While the molecular mechanism by which HDAC inhibition induces mitochondrial ROS in persister cells remains to be determined, the finding that nontoxic concentrations of clinically available HDAC inhibitors specifically sensitize persister cells to ferroptosis highlights another potential combinatorial treatment approach to selectively targeting persister cells for ferroptosis. In addition, hybrid molecules that target both HDACs and induce ferroptosis simultaneously may confer unique responses that warrant additional testing (*65*).

**Fig. 5.**
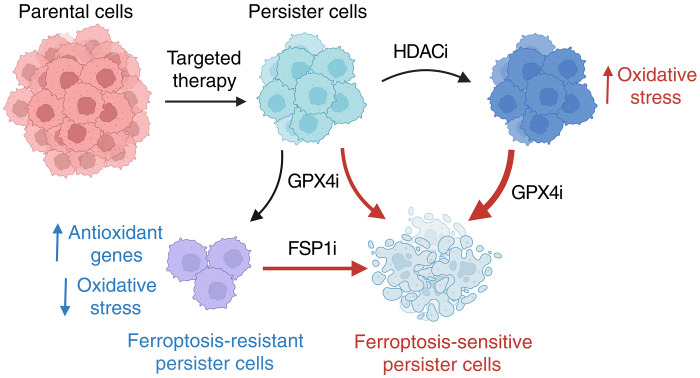
Enhancing persister cell ferroptosis with FSP1 and HDAC inhibition. Cancer persister cells can decrease oxidative stress to survive GPX4 inhibition (GPX4i). However, GPX4i-tolerant persister cells become dependent on the alternative ferroptosis suppressor enzyme FSP1 to survive, and addition of FSP1 inhibitor (FSP1i) increases persister cell ferroptotic death. Furthermore, persister cell oxidative stress is increased by nontoxic pre- or cotreatment with clinically available HDAC inhibitors resulting in synergistic persister cell ferroptosis in combination with GPX4 inhibitor (GPX4i). Our findings reveal previously unexplored approaches to selectively enhancing persister cell ferroptosis. HDACi, histone deacetylase inhibitor. Created in BioRender. Wang, M. (2025) https://BioRender.com/7n2cetj.

Although we have identified persister cell features which affect ferroptosis sensitivity in more than one persister cell type (Fig. 5), a limitation of this study is the analysis of a limited number of persister cell types and drug treatments. Testing of additional persister cell types with different oncogenic signaling and cancer treatments will be required to determine the breadth of relevance of our findings. Furthermore, a fuller understanding of persister cell sensitivity to ferroptosis will require further studies of other features which may differ in persister cells such as phospholipid composition (*17*, *66*), membrane rupture (*67*), and repair (*68*, *69*). Nonetheless, our findings that persister cell ferroptosis is exacerbated by treatment with clinically available HDAC inhibitors or FSP1 inhibition together with GPX4 inhibition reveal previously unexplored potential treatment strategies to eliminate residual disease. Given the concern that GPX4 inhibitors may have unacceptable toxicity in humans (*70*–*72*), discovery of combinatorial approaches such as these may be critical to enable the realization of a clinically effective and safe ferroptosis-based drug treatment for cancer.

## MATERIALS AND METHODS

### Cell culture

PC9 cells were provided by the Altschuler and Wu laboratory at University of California San Francisco. BT474 (HTB-20) and A375 (CRL-1619) cells were purchased from American Type Culture Collection. PC9 cells were cultured in RPMI 1640 (Gibco, 11875093) supplemented with 5% fetal bovine serum (FBS) and 1% antimycotic/antibiotic (AA; Thermo Fisher Scientific). A375 cells were cultured in Dulbecco’s modified Eagle’s medium (high glucose, Gibco, 11965092) supplemented with 10% FBS and 1% AA. BT474 cells were cultured in RPMI 1640 supplemented with 10% FBS and 1% AA. All cells were incubated at 5% CO_2_ and 37°C. Cell lines were split with 0.25% trypsin-EDTA (Thermo Fisher Scientific, 25200056). Cell line identities were confirmed with short tandem repeat (STR) profiling at the UC Berkeley Cell Culture Facility. All cell lines regularly tested negative for mycoplasma throughout these investigations using the Lonza Mycoalert Mycoplasma Detection Kit (Lonza, LT07-318).

### Chemicals

ML210 (SML0521), (1S,3R)-RSL3 (SML2234), decylubiquinone (D7911), buthionine sulfoximine (BSO) (B2515), GSH reduced ethyl ester (CH6H9A56C7FA), (±)-α-tocopherol (258024), and EUK-134 (SML0743) were purchased from Sigma-Aldrich. Lapatinib (S2111), erlotinib (S7786), and iFSP1 (S9663) were purchased from Selleck Chemicals. JQ1 (11187) and panobinostat (13280) were purchased from Cayman Chemical. Vorinostat (HY-10221), dabrafenib (HY-14660), trametinib (HY-10999), metformin (HY-B0627), mitoTEMPO (HY-112879), IACS-010759 (HY-112037), FCCP (HY-100410), and BAY2402234 (HY-112645) were purchased from MedChemExpress. All chemicals except for BSO and metformin, which were dissolved and stored in media, were stored as stock solutions in dimethyl sulfoxide (DMSO; Life Technologies) or cell culture grade water (Corning).

### Persister cell derivation

High-density PC9 and A375 persister cells were used in all experiments unless otherwise noted. BT474 low-density persister cells were used for all experiments. High- and low-density persister cells and density-matched parental cells were derived for each cell line as follows. Low-density PC9 parental cells were seeded at 5200 per well in 96-well plates, and high-density PC9 parental cells were seeded at 21,000 per well in 96-well plates. Low-density A375 parental cells were seeded at 4000 per well in 96-well plates, and high-density A375 parental cells were seeded at 8000 per well in 96-well plates. For PC9 and A375, experiments were performed 24 hours after seeding. Low-density BT474 parental cells were seeded at 3000 per well in 96-well plates, and experiments were performed 72 hours after seeding. To derive PC9 persister cells at low cell density, cells were seeded at 1800 per well in 96-well plates and, after 24 hours, were subjected to continuous treatment with 2.5 μM erlotinib for 10 days. To derive PC9 persister cells at high cell density, cells were seeded at 11,000 per well in 96-well plates and, after 24 hours, were subjected to continuous treatment with 70 nM erlotinib for 10 days unless otherwise stated. To derive A375 persister cells at low cell density, cells were seeded at 2300 per well in 96-well plates and, after 24 hours, were subjected to continuous treatment with 250 nM dabrafenib and 25 nM trametinib for 14 to 15 days. To derive A375 persister cells at high cell density, cells were seeded at 4000 per well in 96-well plates and, after 24 hours, were subjected to continuous treatment with 10 nM dabrafenib and 1 nM trametinib for 14 to 15 days. To derive BT474 low-density persister cells, cells were seeded at 5500 per well in 96-well plates and, after 72 hours, were subjected to continuous treatment with 2 μM lapatinib for 10 days. For all persister cell drug treatments, targeted therapy was refreshed every 3 to 4 days.

### Cell viability assays

Cell viability was evaluated by measuring ATP levels using CellTiter-Glo Luminescent Cell Viability assay (CTG) (Promega, G7570) according to the manufacturer’s manual. Sample luminescence was measured in black optical-bottom plates (Corning, 39304) using the SpectraMax iD3 microplate reader and SoftMax Pro 7 Software.

### scRNA-seq analysis

To derive persister cells for the scRNA-seq experiments, PC9 cells were adhered overnight and then treated with 100 nM erlotinib for 10 days. Parental PC9 cells were seeded on day 9 of persister cell treatment. The following day, or following persister cell derivation, 1 μM RSL3 was added to both persister and parental cells, and samples were collected for single-cell library preparation after 24 hours in RSL3. For panobinostat treatment, parental and persister cells were treated with 7.5 nM panobinostat for 48 hours before collection. Cells were analyzed with the 10x Genomics 3’ Chromium v3 platform for scRNA-seq according to the manufacturer’s protocol. Libraries were generated and checked for quality and concentration using an Agilent Tapestation and Qubit, respectively. Samples were combined and sequenced using an Illumina NovaSeq 6000 (Flow Cell Type: S4).

### scRNA-seq data mapping and processing

The Cell Ranger Single-Cell Software Suite (version 3.1.0) was used to align fastq files to the human reference genome “refdata-cellranger-GRCh38-3.0.0” with the “cellranger count” command. Mapped reads from individual samples were then merged as Seurat objects (Seurat version 3.1) (*73*, *74*). We selected cells with greater than 1000 and less than 7500 features and with less than 20% mitochondrial content for downstream analysis. Normalization and scaling were performed with the Seurat “SCTransform” command with cell cycle gene regression (*75*, *76*). Downstream commands “RunPCA,” “RunUMAP,” “FindNeighbors,” and “FindClusters” were performed with default settings, with 30 dimensions used for RunUMAP and FindNeighbors. Differentially expressed genes were calculated with the Seurat “FindMarkers” command without expression or cell number thresholds. Gene set enrichment analysis was conducted with the ClusterProfiler R package (version 3.18.0) with default settings using Hallmarks and oncogenic signature gene sets (*77*). Pseudotime analysis was performed with Slingshot (version 2.14.0) with both persister cell clusters set as the starting clusters (*78*).

### Seahorse metabolic assay

For persister derivation, PC9 cells were seeded at 11,000 cells per well in XFe96 Cell Culture Microplate (Agilent, #103794-100). Erlotinib (70 nM) was added the next day and maintained for 12 days with media changes every 3 days. For chronic metformin-treated wells, 2.5 mM metformin was added and maintained with 70 nM erlotinib and refreshed with media changes. Panobinostat (7.5 nM) was added 2 days before assay. RSL3 (500 nM) was added 1 day before assay. For the parental population, PC9 cells were seeded at 10,000 cells per well 1 day before assay. Seahorse media was prepared using phenol-free XF base media supplemented with 17.5 mM glucose, 2 mM GlutaMAX (glutamine), and 0.5 mM pyruvate. The oxygen consumption rate of all conditions was measured using the XFe96 respirometer under basal conditions and after injection of the following: 1.25 μM oligomycin, 1 μM FCCP, and 1 μM rotenone with 1 μM antimycin A. Data were analyzed using Wave Software (v2.6.1).

### Immunoblotting

Parental and persister cells were washed with phosphate-buffered saline and lysed with radioimmunoprecipitation assay (RIPA) buffer (Thermo Fisher Scientific, 89900) with a phosphatase inhibitor (Thermo Fisher Scientific, 78420) and a protease inhibitor (Thermo Fisher Scientific, 78430). Lysates were centrifuged at 14,000*g* at 4°C for 15 min, and protein concentration was determined using the Pierce BCA Protein Assay Kit (Thermo Fisher Scientific, 23225). Lysates were combined with sample buffer (Thermo Fisher Scientific, NP0007) and incubated at 70°C for 10 min. Samples were run on SDS–polyacrylamide gel electrophoresis gels (Bolt 4 to 12% Bis-Tris Gel, Life Technologies, NW04120BOX), with the Chameleon Duo Pre-stained Protein Ladder (LI-COR, #928-60000), and transferred to a nitrocellulose membrane using the iBLOT 2 Dry Blotting System (Life Technologies, IB21001). Membranes were blocked with 5% bovine serum albumin (GeminiBio, 700-100P-1KG) for 1 hour at room temperature before overnight incubation at 4°C. The following day, LI-COR secondary antibodies were incubated with the membrane for 1 hour at room temperature, and membranes were imaged using the LI-COR Odyssey Imaging System and Image Studio version 5.2. Loading controls were either β-tubulin or vinculin as indicated. Antibody commercial sources were as follows: β-tubulin (Invitrogen, MA5-16308), vinculin (Cell Signaling Technology, #4650), NRF2 (Cell Signaling Technology, #12721), KEAP1 (Cell Signaling Technology, #8047), SLC7A11 (Cell Signaling Technology, #12691), SLC3A2 (Cell Signaling Technology, #47213), GPX4 (Cell Signaling Technology, #59735), AIFM2/FSP1 (Cell Signaling Technology, #51676), DHODH (Cell Signaling Technology, #26381), VKORC1L1 (Cell Signaling Technology, #29458), phosphorylated AMPK (Cell Signaling Technology, #2535), total AMPK (Cell Signaling Technology, #5831), IRDye 680RD goat anti-mouse immunoglobulin G (IgG) secondary antibody (LI-COR, #926-68070), and IRDye 800CW goat anti-rabbit IgG secondary antibody (LI-COR, #926-32211).

### GSH and ATP measurements

Intracellular levels of total GSH were measured using GSH-Glo Glutathione Assay (Promega, V6911) according to the Assay Procedure for Adherent Mammalian Cells in the manufacturer’s manual. ATP levels were measured using CTG (Promega, G7570). Both measurements were normalized by protein levels measured by the Pierce BCA Protein Assay Kit (Thermo Fisher Scientific, 23225) after protein isolation from cells using RIPA lysis and extraction buffer as previously described (Thermo Fisher Scientific, 1338-43-8).

### Measurement of synergistic drug effects

Cells plated in 96-well plates were treated with six concentrations of each chemical. After treatment with the respective chemicals for 24 hours, cell viability was quantitated by CellTiter-Glo. Bliss synergy scores were calculated using SynergyFinder 3.0 (*79*).

### Intracellular ROS measurements

The intracellular ROS level of persister cells was quantitated by flow cytometry using the CellROX Deep Red Flow Cytometry Assay Kit (Life Technologies, C10491) for total ROS and mitoSOX Red (Thermo Fisher Scientific, M36008) for mitochondrial ROS, according to the manufacturer’s manual. Briefly, persister cells were treated with 7.5 nM panobinostat or DMSO for 2 days under continuous erlotinib treatment and then subjected to flow cytometry. For a CellROX-positive control, cells were treated with 200 μM tert-butyl hydroperoxide for 60 min before the flow cytometry assay. Trypsin-lifted cells were incubated with 1 μM CellROX Deep Red or 1 μM mitoSOX Red reagent for 60 min at 37°C without light exposure. During the final 15 min of staining, 1 μM SYTOX Blue Dead Cell stain (Thermo Fisher Scientific, S34857) solution was added to cells, followed by immediate analysis by flow cytometry. See fig. S4 for the mitoSOX gating strategy and fig. S8 for the CellROX gating strategy.

### Iron measurement

The Cell Ferrous Iron Colorimetric Assay Kit (Elabscience, E-BC-K881-M) was used to detect intracellular labile ferrous iron (Fe^2+^) according to the manufacturer’s manual. PC9 parental and persister cells were treated with 7.5 nM panobinostat or DMSO control for 2 days. Cells were then trypsinized and counted to retrieve 5 million cells per replicate. Background measurement from a control solution was subtracted from each sample, and iron concentrations were calculated from a standard curve.

### Statistical analyses

Statistical tests and graphing were performed with GraphPad Prism 9.3.1 except for synergy calculation which was performed by SynergyFinder 3.0 (*79*). Unless otherwise noted, *P* values were calculated using unpaired, two-tailed *t* tests assuming unequal variance. For ferroptosis driver and ferroptosis suppressor gene set signature scoring comparisons, *P* values were calculated with the Mann-Whitney test. All experiments were performed at least two times except for fig. S4B and the scRNA-seq experiments.

## Supplementary Materials

### The PDF file includes:

Figs. S1 to S8 Uncropped Western blot images Legends for tables S1 to S8
