# Supplementary material for: FSP1 and histone deacetylases suppress cancer persister cell ferroptosis
Source: Sci Adv. 2026 Jan 2;12(1):eaea8771. doi: 10.1126/sciadv.aea8771 (PMC12758554; doi:10.1126/sciadv.aea8771)
Supplement: Supplementary file 1 — Figs. S1 to S8 Uncropped Western blot images Legends for tables S1 to S8 [file sciadv.aea8771_sm.pdf]

Science Advances
AAAS

Supplementary Materials for

FSP1 and histone deacetylases suppress cancer persister cell ferroptosis

Masayoshi Higuchi *et al.*

Corresponding author: Matthew J. Hangauer, mhangauer@ucsd.edu

Sci. Adv. **12**, eaea8771 (2026)
DOI: 10.1126/sciadv.aea8771

The PDF file includes:

Figs. S1 to S8
Uncropped Western blot images
Legends for tables S1 to S8

Other Supplementary Material for this manuscript includes the following:

Tables S1 to S8

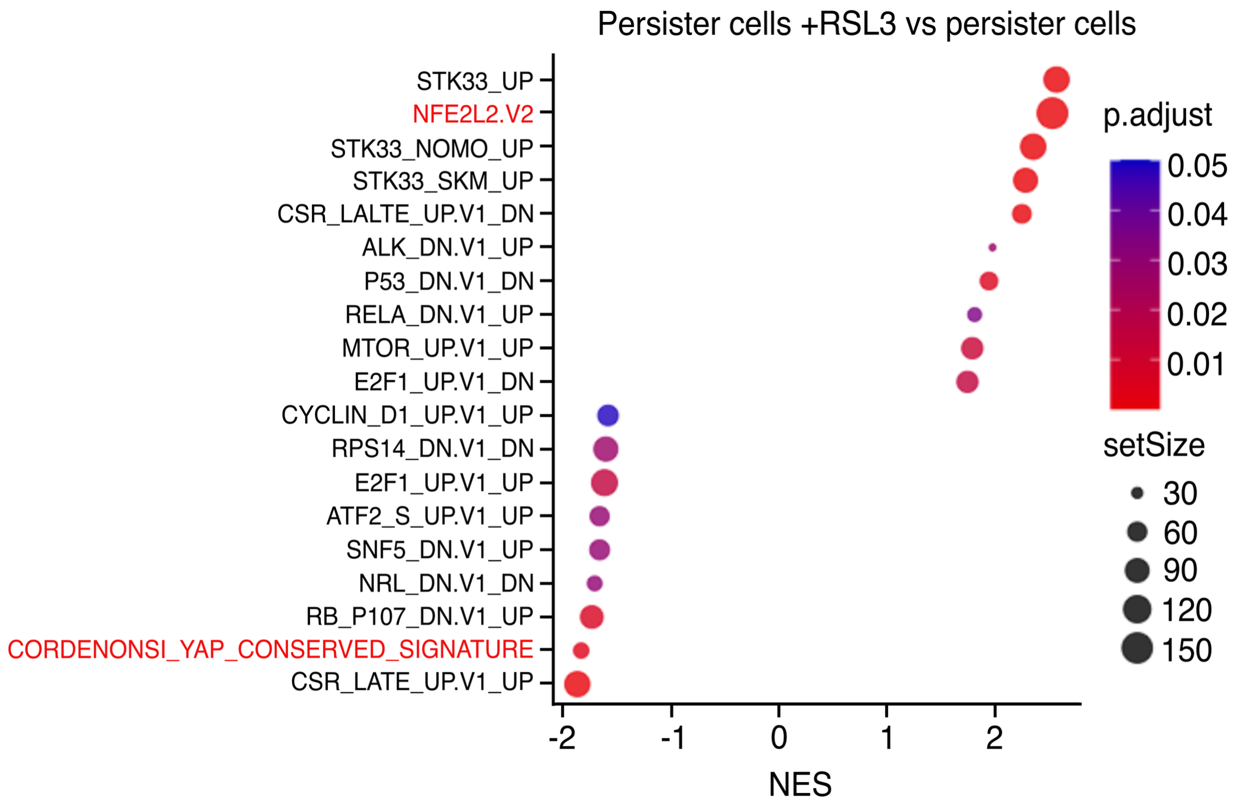

Fig. S1. Enriched Oncogenic Signature gene sets between persister cells treated with or without RSL3. Positive Normalized Enrichment Score (NES) values indicate increased gene set expression in cells treated with RSL3. NFE2L2.V2 refers to the NRF2 gene set.

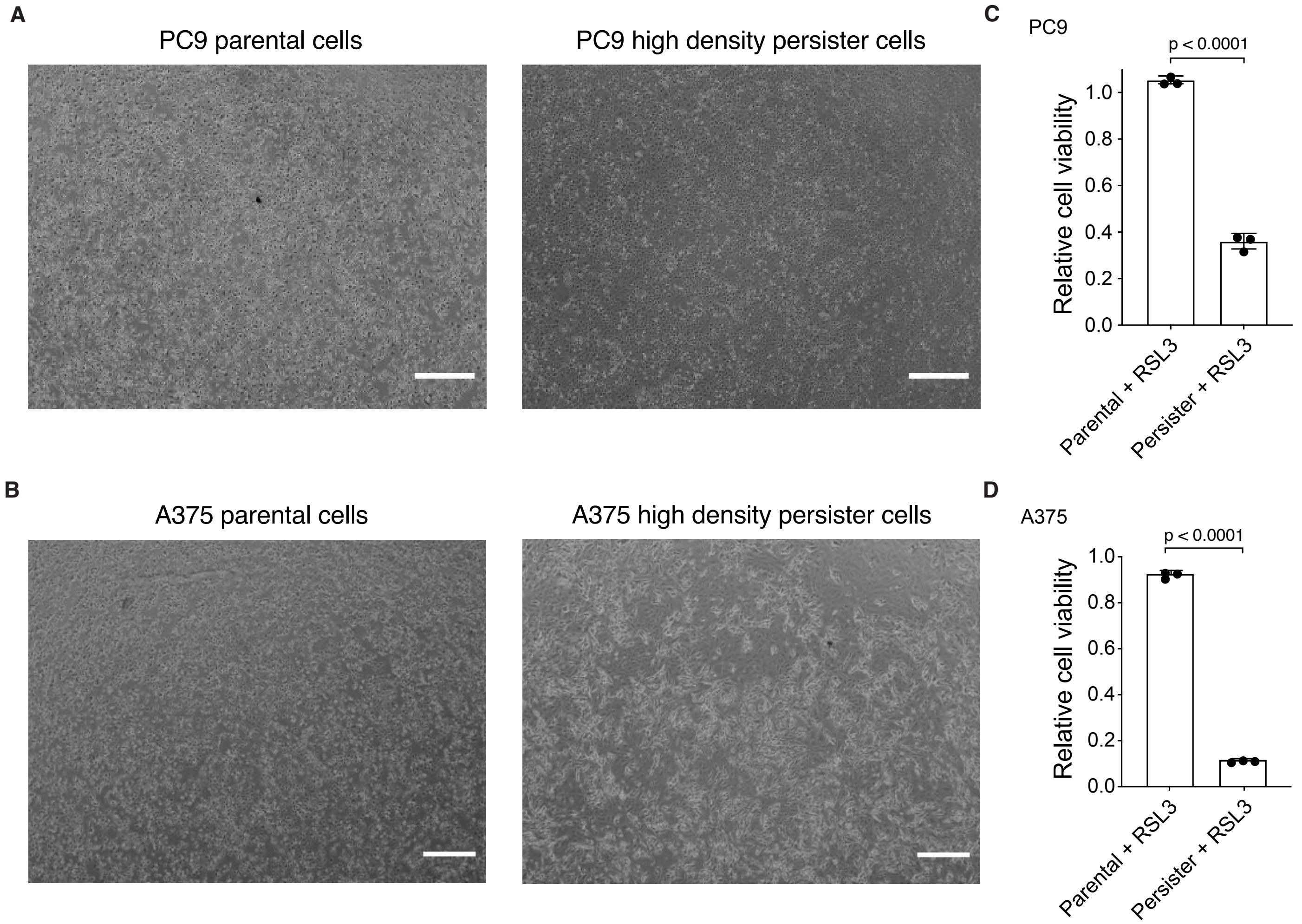

Fig. S2. Persister cells remain sensitized to ferroptosis at high confluency. (**A** and **B**) High density persister cells derived from 70 nM erlotinib for PC9 cells and 10 nM dabrafenib and 1 nM trametinib for A375 cells. Parental cells of similar density are also shown. See Materials and Methods. (**C** and **D**) Cell viability of PC9 and A375 high density persister cells and density-matched parental cells treated with 1 µM RSL3 for 24 hours. n = 3 biological replicates; mean ± s.d. is shown; P values calculated with two-tailed Student's t-test. Scale bar is 100 µm.

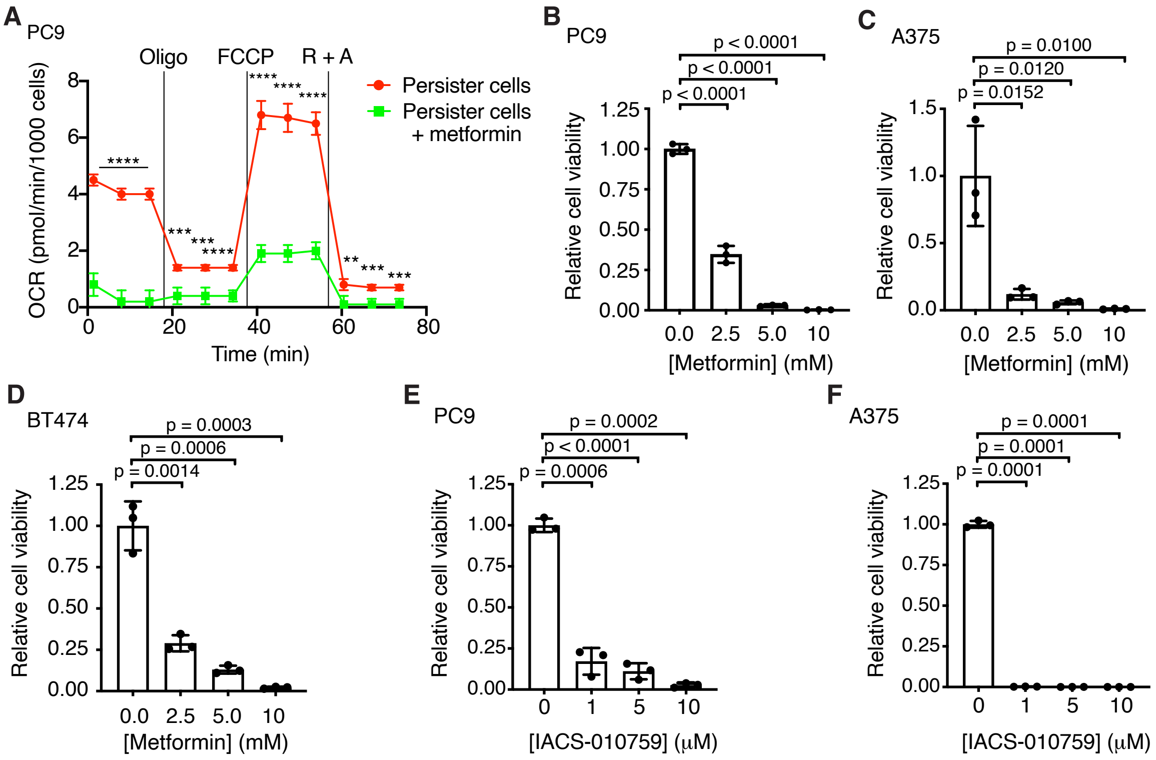

Fig. S3. Persister cells are sensitive to electron transport chain inhibition. (**A**) PC9 persister cells from 70 nM erlotinib derived with and without 2.5 mM metformin were analyzed for oxygen consumption rate (OCR). 1.25 μM Oligomycin (Oligo), 1 μM FCCP, and 1 μM rotenone plus 1 μM antimycin A (R+A). *n* = 3 biological replicates; mean ± s.e.m. is shown; **P<0.01, ***P<0.001, ****P<0.0001. (**B** to **D**) PC9 persister cells derived from treatment with 2.5 μM erlotinib (**B**) A375 persister cells derived from 250 nM dabrafenib and 25 nM trametinib (**C**), and BT474 persister cells derived from 2 μM lapatinib (**D**) were cotreated with the indicated metformin concentrations throughout drug treatment. (**E** to **F**) PC9 persister cells derived with 2.5 μM erlotinib (**E**) and A375 persister cells derived from 250 nM dabrafenib and 25 nM trametinib (**F**) were cotreated with the indicated IACS-010759 concentration throughout drug treatment. (**B** to **F**) *n* = 3 biological replicates; mean ± s.d. is shown; P values calculated with two-tailed Student's t-test.

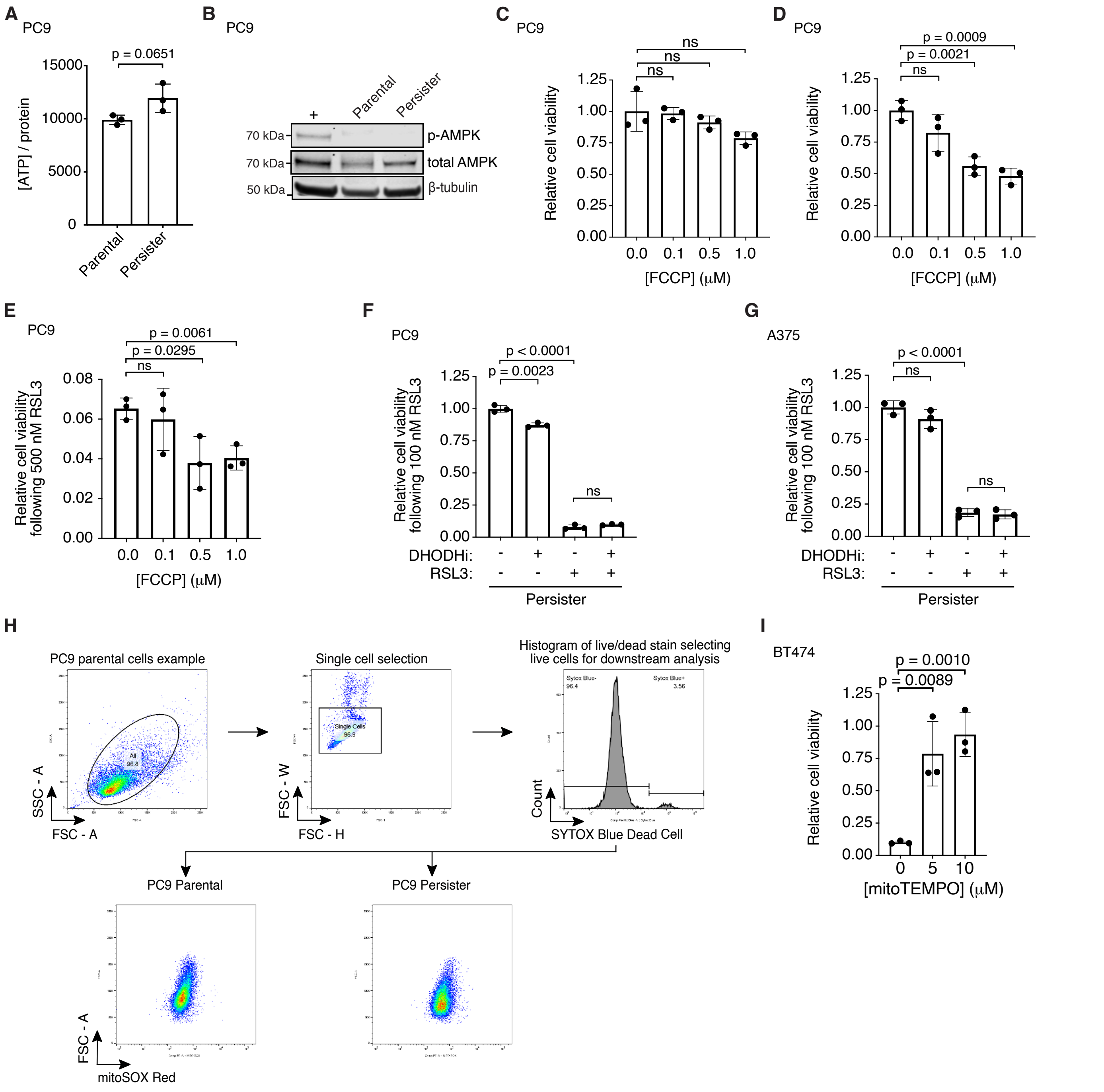

Fig. S4. Persister cell ferroptosis sensitivity is not driven by ATP production or enhanced by DHODH inhibition. (**A**) ATP concentration in parental and persister cells derived from 2.5 μM erlotinib. (**B**) Western blot for phosphorylated and total AMPK levels. Positive control (+) is 24 hour treatment with 5 mM metformin. Persister cells were derived with 2.5 μM erlotinib. (**C** and **D**) PC9 parental (**C**) and persister cells derived from 2.5 μM erlotinib (**D**) were treated with the indicated FCCP concentration for 48 hours and normalized to their respective population without FCCP. (**E**) Persister cells were treated with the indicated FCCP concentration for 48 hours and with 500 nM RSL3 for 24 hours. Cell viability was normalized to the viability of the respective FCCP-treated persister cells without RSL3 to account for FCCP toxicity. (**F** and **G**) PC9 (**F**) and A375 (**G**) persister cells derived from 2.5 μM erlotinib and 250 nM dabrafenib and 25 nM trametinib, respectfully, were

treated with 100 nM DHODH inhibitor BAY2402234 for 24 hours with or without 100 nM RSL3. (**H**) Flow cytometry gating strategy for mitochondrial ROS. Persister cells derived from 2.5 μM erlotinib. (**I**) BT474 persister cells derived from 2 μM lapatinib were treated with mitoTEMPO for 72 hours and then co-treated with 500 nM RSL3 for 24 hours. Viability was normalized to the respective mitoTEMPO-treated persister cells without RSL3 treatment. (**A**, **C**-**G,** and **I**) n = 3 biological replicates; mean ± s.d. is shown; P values calculated with two-tailed Student's t-test.

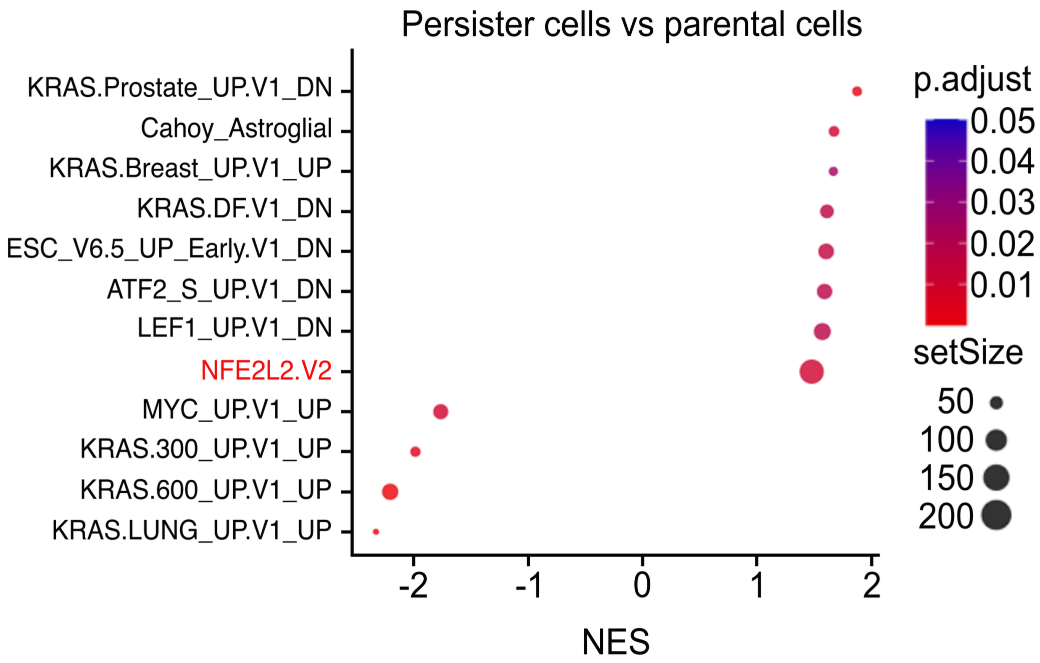

Fig. S5. Enriched Oncogenic Signatures gene sets between PC9 parental and persister cells. Positive Normalized Enrichment Score (NES) values indicate gene sets upregulated in persister cells. NFE2L2.V2 refers to the NRF2 gene set.

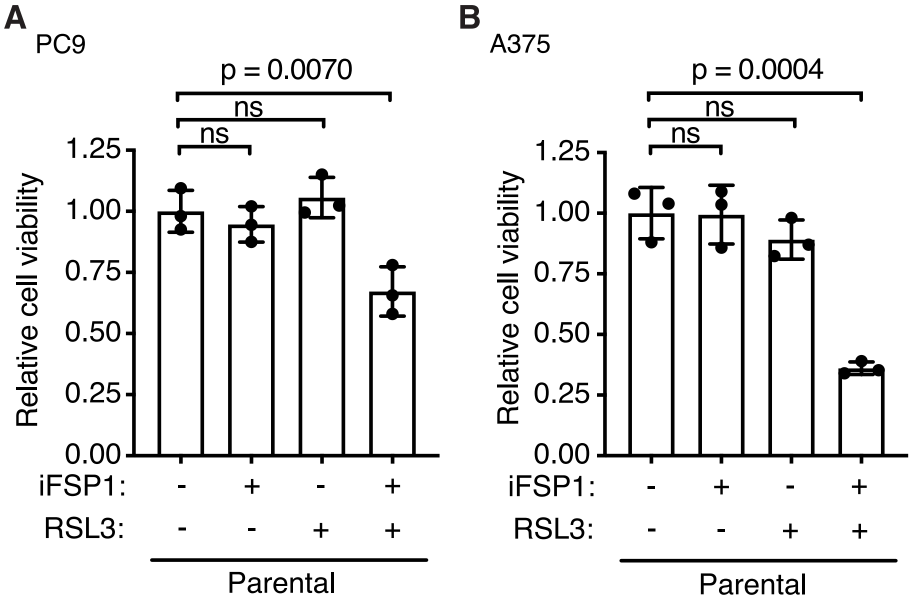

Fig. S6. FSP1 and GPX4 inhibitor treatment of parental cells. (**A** and **B**) PC9 (**A**) and A375 (**B**) parental cells treated with 1 μM FSP1 inhibitor, 50 nM RSL3, or both in combination for 24 hours. *n* = 3 biological replicates; mean ± s.d. is shown; P values calculated with two-tailed Student's t-test.

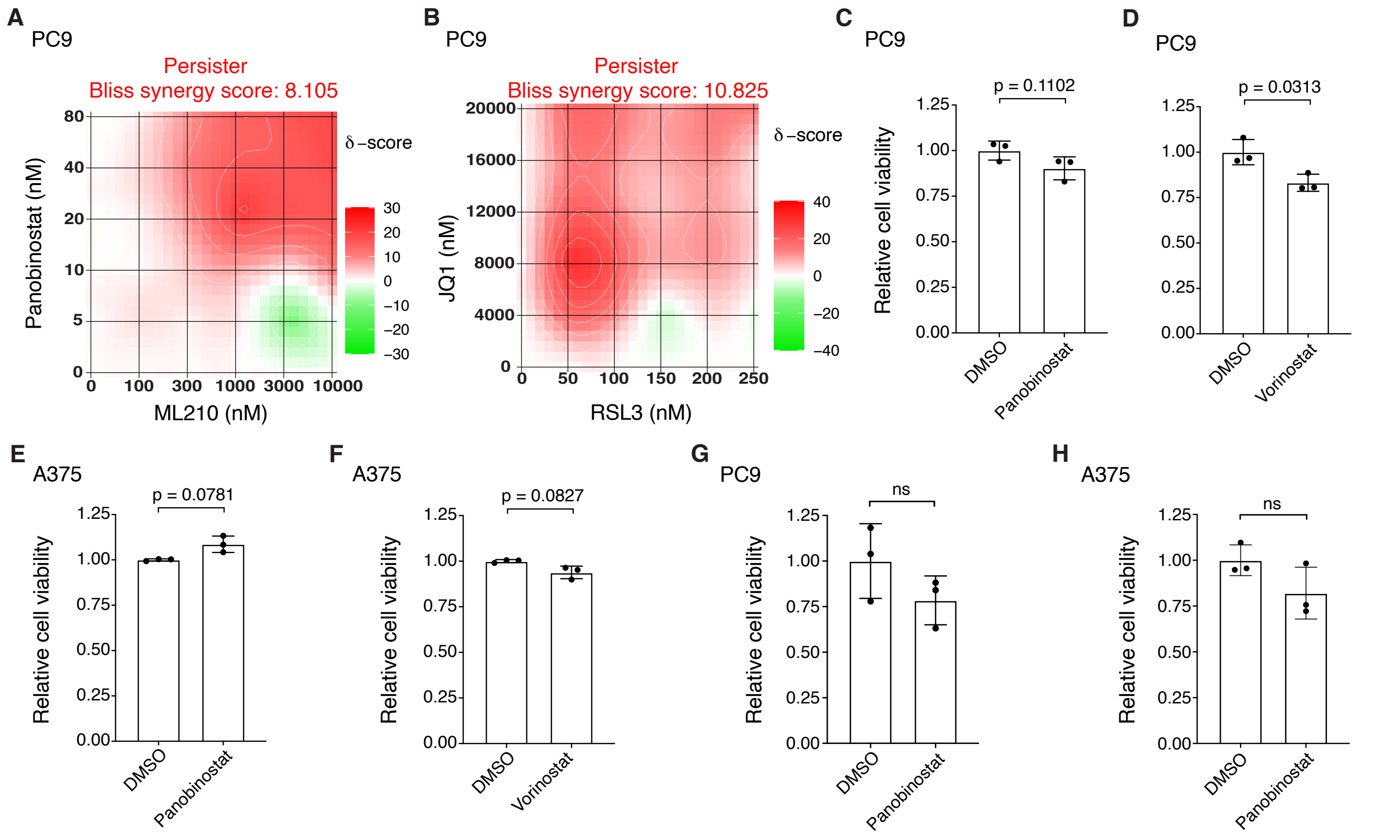

Fig. S7. Additional data demonstrating HDAC and BRD4 inhibitors synergize with GPX4 inhibitors to kill persister cells. (**A** and **B**) Heatmaps of synergy between GPX4 inhibitors ML210 (**A**) or RSL3 (**B**) and HDAC inhibitor panobinostat (**A**) or BRD4 inhibitor JQ1 (**B**) following 24 hour cotreatment of pre-derived PC9 persister cells. Bliss synergy score calculated with SynergyFinder 3.0. (**C** to **F**) Persister cells are insensitive to HDAC inhibitor concentrations which sensitize persister cells to ferroptosis. PC9 and A375 persister cell viability was measured following 48 hour treatment with 7.5 nM panobinostat (**C**), 100 nM vorinostat (**D**), 5 nM panobinostat (**E**), or 1 µM vorinostat (**F**). (**G** and **H**) PC9 (**G**) and A375 (**H**) parental cells were treated with 7.5 nM or 5 nM panobinostat, respectively, for 48 hours prior to washout and treatment for 24 hours with 150 nM (**G**) or 100 nM (**H**) RSL3. (**C-H**) $n = 3$ biological replicates; mean ± s.d. is shown; P values calculated with two-tailed Student's t-test.

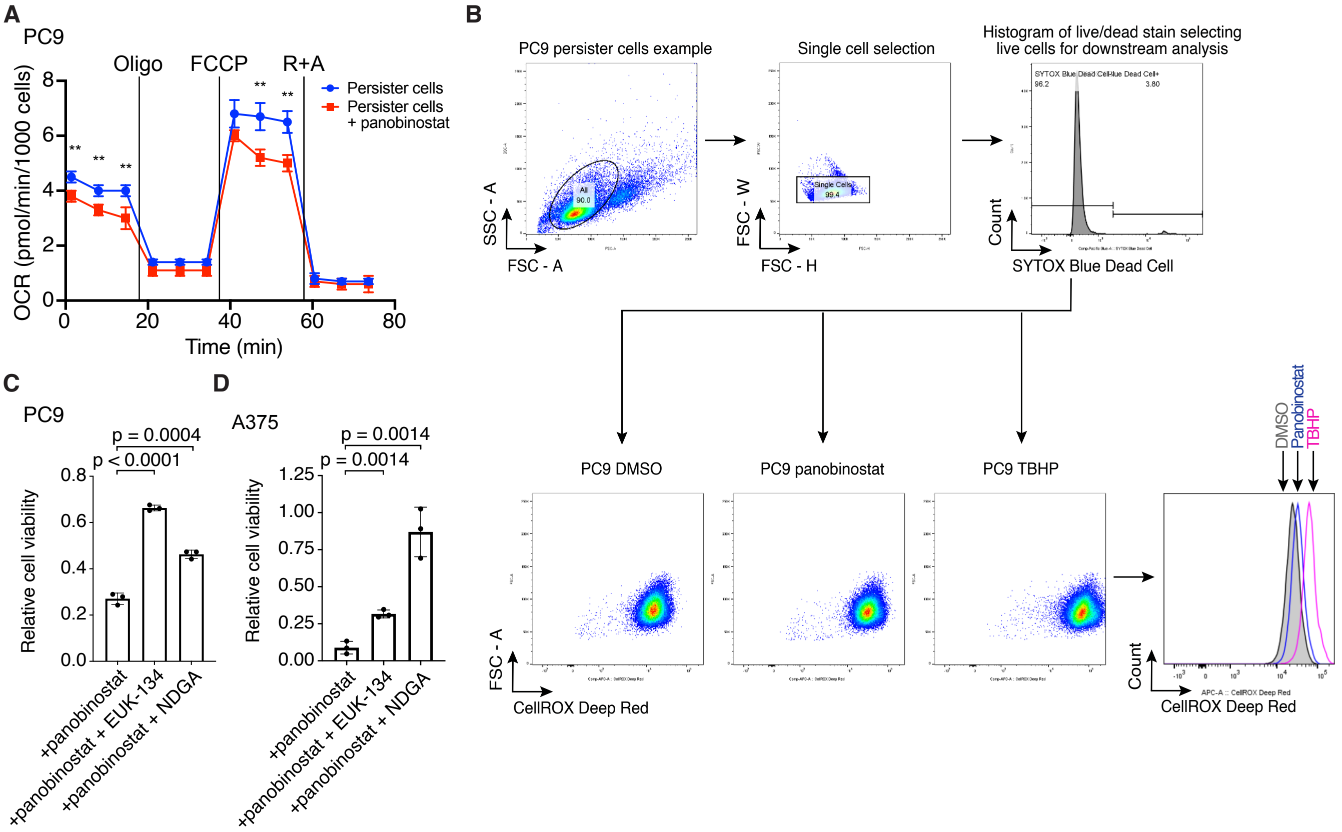

Fig. S8. HDAC inhibitors increase ROS to sensitize persister cells to ferroptosis. (**A**) PC9 persister cells, and persister cells treated with 7.5 nM panobinostat for 48 hours were analyzed for oxygen consumption rate (OCR). 1.25 µM Oligomycin (Oligo), 1 µM FCCP, and 1 µM rotenone plus 1 µM antimycin A (R+A) were used. *n* = 3 biological replicates; mean ± s.e.m. is shown; P values calculated between conditions using two-tailed Student's t-test. **P<0.01. (**B**) Gating strategy used for Fig. 4G, where geometric means of CellROX deep red signal from live, single cells are graphed. PC9 persister cells were co-treated with DMSO or 7.5 nM panobinostat for 48 hours. Positive control cells were treated with 200 µM tert-butyl hydroperoxide (TBHP) for 1 hour. (**C** and **D**) PC9 (**C**) and A375 (**D**) persister cells treated with 2.5 nM panobinostat are rescued from RSL3 treatment with the antioxidants EUK-134 (10 µM) and nordihydroguaiaretic acid (NDGA, 5 µM). *n* = 3 biological replicates; mean ± s.d. is shown; P values calculated with two-tailed Student's t-test.

Uncropped western blot images

For uncropped western blot images, red boxes indicate bands shown in the main figure while blue boxes indicate loading controls that were not included in the main figures.

Fig. S4B

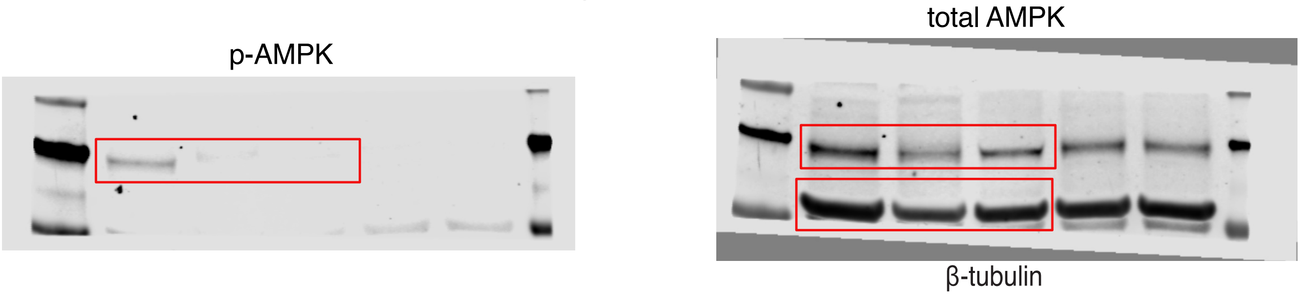

Fig. 2A

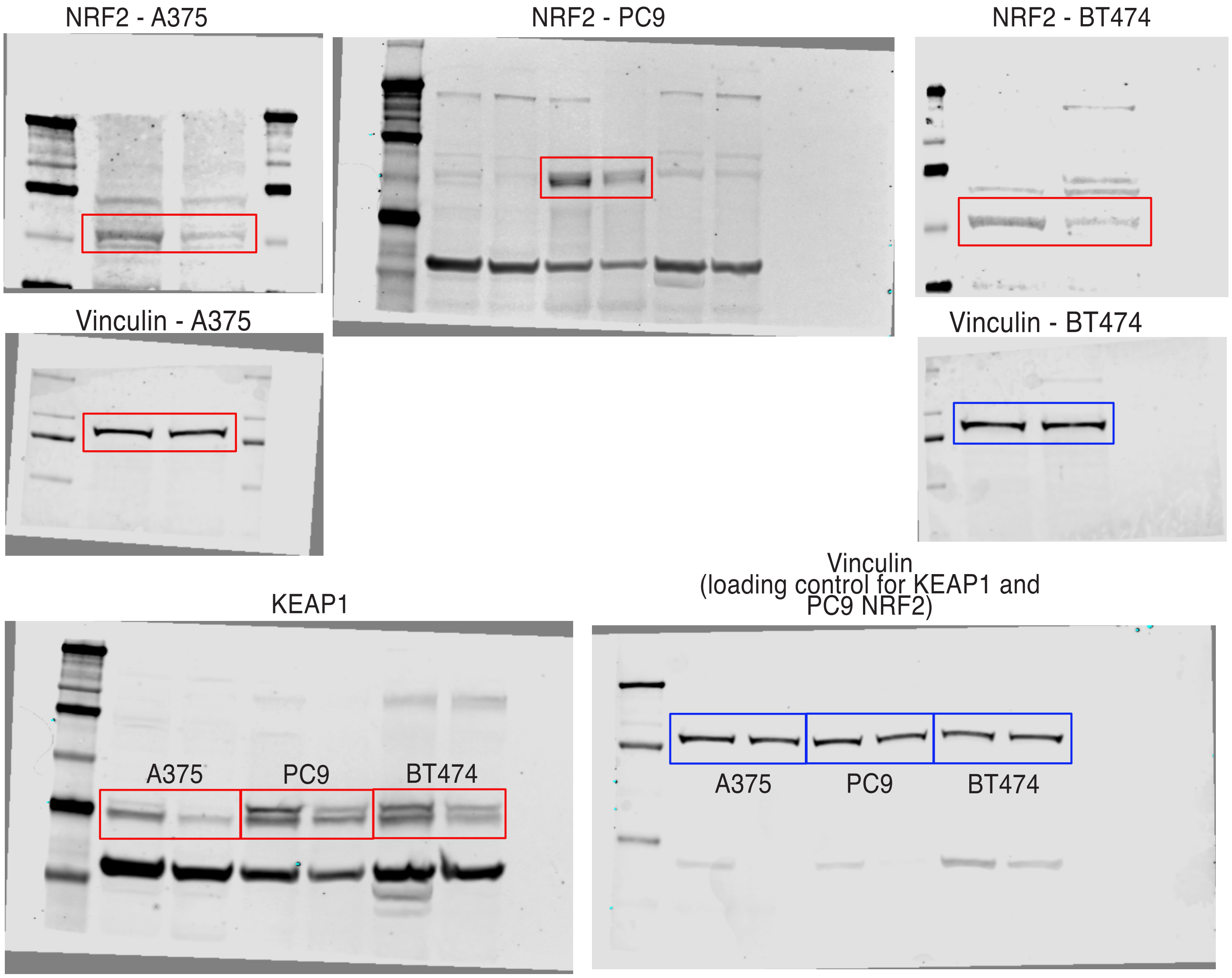

Fig. 2A (continued)

β-tubulin (loading control for SLC7A11 and SLC3A2)

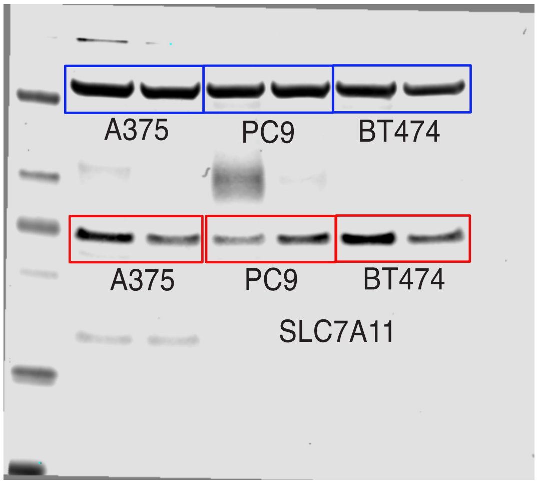

SLC3A2

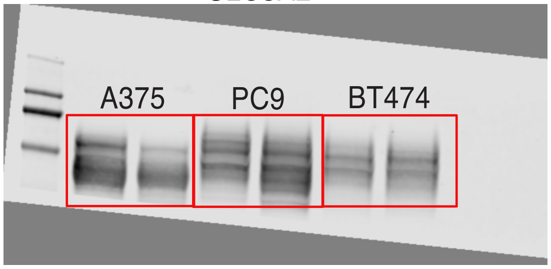

Fig. 2D

FSP1 - A375

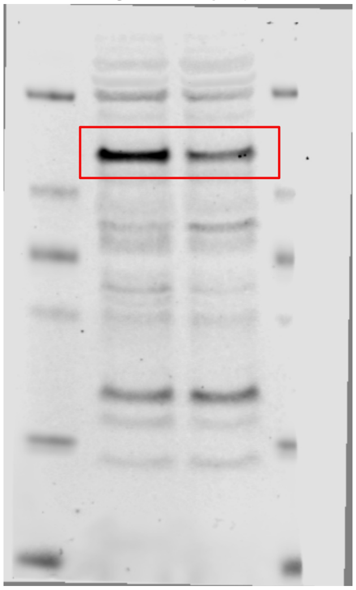

FSP1 - PC9

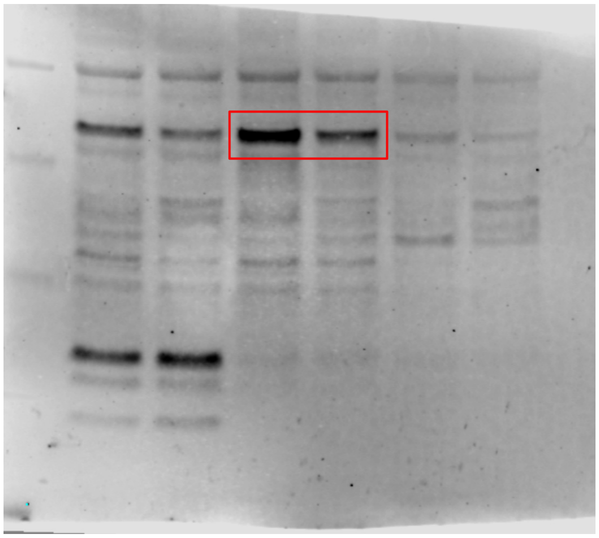

FSP1 - BT474

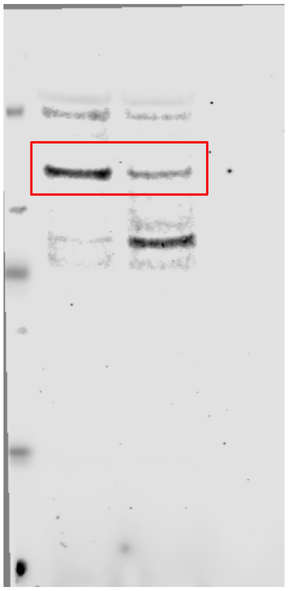

β-tubulin - A375

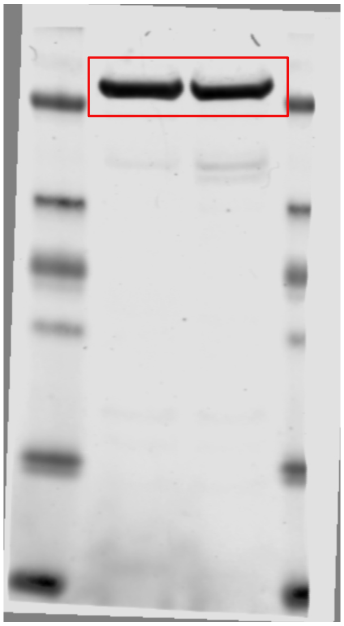

β-tubulin - PC9

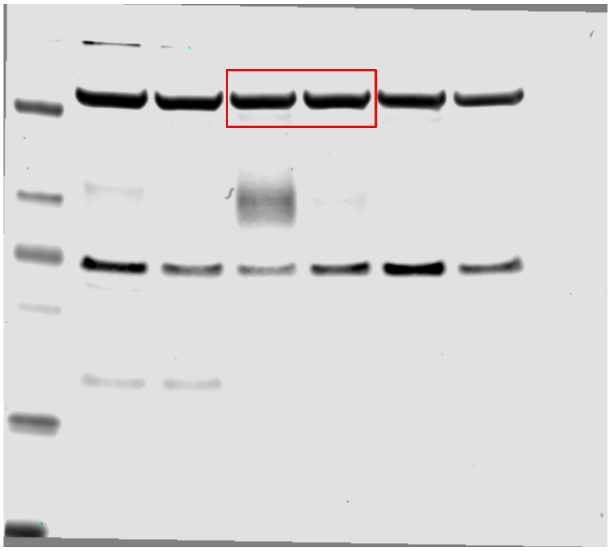

β-tubulin - BT474

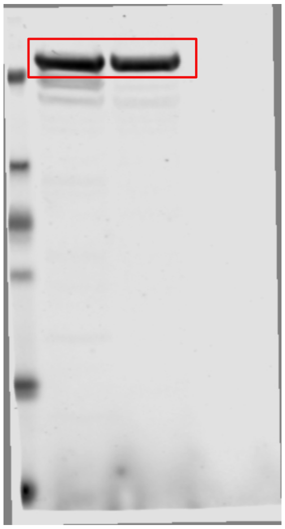

Fig. 2D (continued)

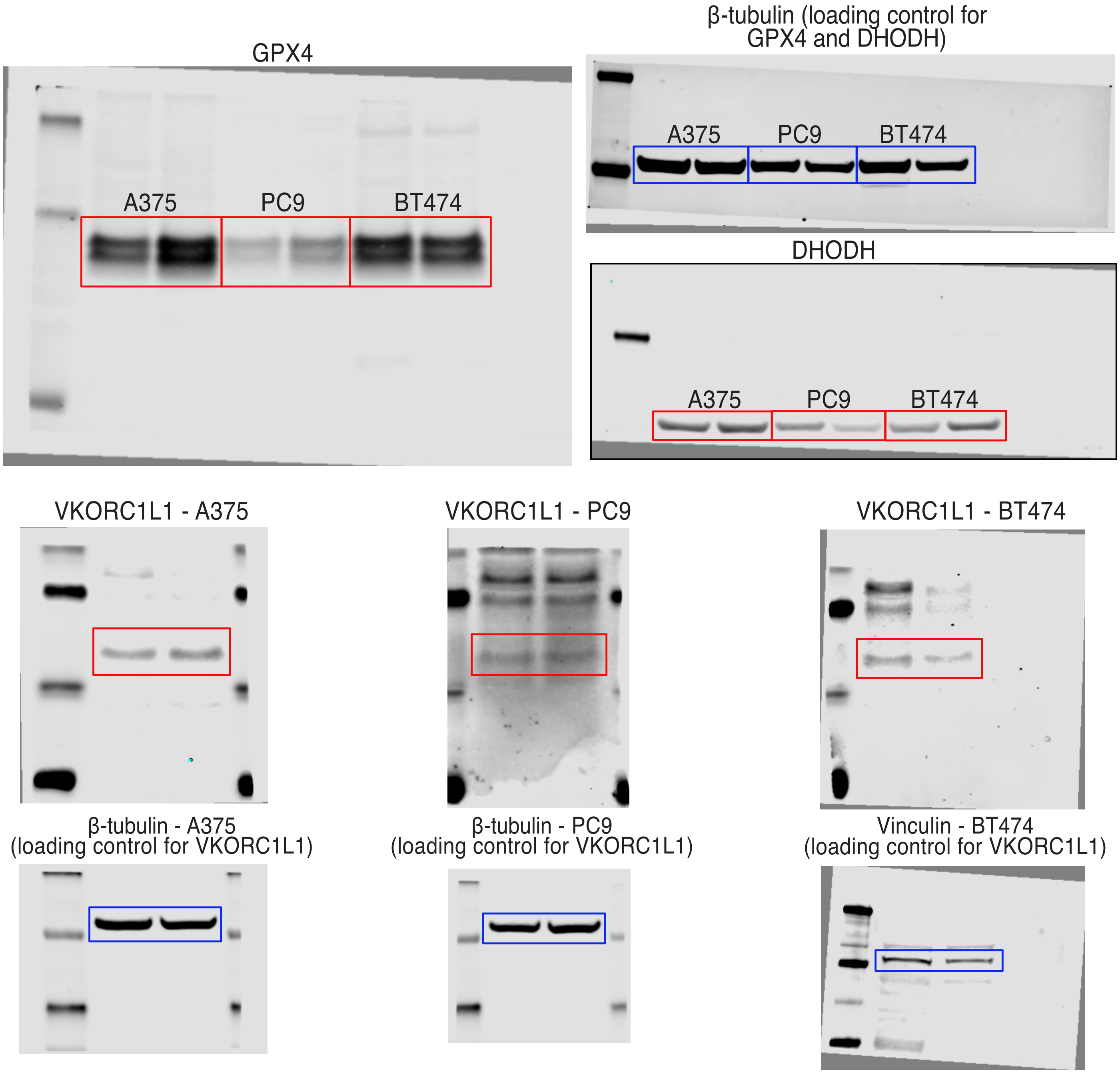

Captions for Tables S1-S8

Table S1. Differentially expressed genes between PC9 persister cells treated with RSL3 versus without RSL3.

Table S2. Enriched Hallmarks and Oncogenic Signatures gene sets between PC9 persister cells treated with RSL3 versus without RSL3.

Table S3. Differentially expressed genes between PC9 persister cell clusters treated with RSL3 versus without RSL3.

Table S4. Enriched Hallmarks and Oncogenic Signatures gene sets between PC9 persister cell clusters treated with RSL3 versus without RSL3.

Table S5. Differentially expressed genes between PC9 persister and parental cells.

Table S6. Enriched Hallmarks and Oncogenic Signatures gene sets between PC9 persister and parental cells.

Table S7. Differentially expressed genes between PC9 persister cells treated with panobinostat versus without panobinostat.

Table S8. Enriched Hallmarks gene sets between PC9 persister cells treated with panobinostat versus without panobinostat.
